# Current Evidence and Future Perspectives of the Best Supplements for Cardioprotection: Have We Reached the Final Chapter for Vitamins?

**DOI:** 10.31083/j.rcm2311381

**Published:** 2022-11-09

**Authors:** Farah Yasmin, Syed Hasan Ali, Aisha Naeem, Subhan Savul, Muhammad Sohaib Iqbal Afridi, Neha Kamran, Fawwad Fazal, Shehryar Khawer, Ilma Saleh Savul, Hala Najeeb, Hamdoon Suharwardy Asim, Marium Nausherwan, Muhammad Sohaib Asghar

**Affiliations:** ^1^Department of Internal Medicine, Dow Medical College, Dow University of Health Sciences, 74200 Karachi, Pakistan; ^2^Department of Internal Medicine, Ziauddin University, 75000 Karachi, Pakistan; ^3^Department of Internal Medicine, St Joseph Medical Center, Houston, TX 77002, USA; ^4^Division of Nephrology and Hypertension, Mayo Clinic, Rochester, MN 55902, USA

**Keywords:** cardiovascular disease, atherosclerosis, endothelial dysfunction, arterial hypertension, vitamin supplementation

## Abstract

Cardiovascular disease (CVD), a broad-spectrum term comprising coronary artery disease, stroke, hypertension, and heart failure, presents as one of the most significant strains on global healthcare systems. Coronary artery disease, caused by atherosclerosis, has various modifiable risk factors such as dietary changes and exercise. Since these risk factors are found to be linked to oxidative stress and inflammations, the dietary supplementation with vitamins’ role in treating and preventing the diseases has been of much debate. With various vitamins having anti-inflammatory and antioxidative properties, studies have explored their correlation with cardiovascular health. Therefore, this narrative review explores and evaluates the benefits and risks of all vitamin supplementations in patients with CVD and provides future recommendations.

## 1. Introduction

Cardiovascular disease (CVD) is one of the major causes of morbidity and mortality worldwide. In 2020, approximately 690,882 people died from CVDs, representing a significant burden on global mortality [1]. Amongst deaths caused by CVDs, 43.8% of the deaths are contributed by coronary artery disease (CAD), followed by stroke, high blood pressure, and heart failure (HF) [2]. Atherosclerosis is the chief etiologic process behind CADs, with its progression related to an interplay between genetic and environmental risk factors [3, 4, 5, 6, 7]. Many of the risk factors of CVDs are presumed to increase the process by promoting inflammation and oxidative stress. Micronutrients, including vitamins, are thought to play a significant role in pathways that lower inflammation and oxidative damage and thus are thought to play a role in reducing CVD risk.

The rationale for taking oral multivitamins is that they promote good health, including the prevention of CVD. Up to 52% of the United States’ (US) population takes dietary supplements which includes multivitamins in 31% of the population, vitamin D in 19%, calcium in 14%, and vitamin C supplement which has been reported as high as 52% in various surveys [8, 9]. The uncertainty of whether individuals would benefit from vitamin supplementation has been the subject of numerous investigations. According to the US Preventive Services Task Force (USPSTF), the existing data supporting the use of multivitamins or other nutrient supplements to prevent cardiovascular illnesses is poor [10, 11, 12]. This narrative study review assesses the risks and benefits of the most common vitamin supplements used for cardiovascular health.

## 2. Effects of Vitamin Supplementation on Cardiovascular Disease

### 2.1 Vitamin A

Preformed vitamin A (retinol and retinyl esters) and provitamin A carotenoids, such as beta-carotene, which are converted to retinol, are the two main forms of vitamin A in the human diet [13]. It is believed to be involved in angiogenesis, oxidative balance, and cellular development, thus, influencing the cardiovascular system and metabolic disorders [14].

Retinoids, vascular responses to damage, and atherogenesis are all related. Proteins involved in vitamin A metabolism, including retinol-binding protein 4 (RBP4) and aldehyde dehydrogenase 1A1 have been to be associated with metabolic disease [15]. After four days of vitamin A administration, animal studies showed a significant reduction in atheromatous plaque and a decrease in intimal damage via changing the migration and proliferation of smooth muscle cells, which play a crucial role in atherogenesis [16]. According to Noy *et al*. [17], Vitamin A metabolite all-*trans*-retinoic acid (RA) regulates growth, remodeling, and metabolic responses in adult tissues. On a cellular level, all trans retinoic acid inhibits neointima formation in endothelial cells by inducing Plasminogen activator inhibitor-1 (PAI-1) [18]. Another study [19] demonstrates that retinoid receptors enhance intracellular adhesion molecule-1 (ICAM-1) protein expression in endothelial cells which is involved in a number of pathologic conditions including atherosclerosis and stroke. There is evidence to support the theory that increased lutein levels are responsible for the protection provided by vegetables and fruits against CVD. It has antioxidant qualities and stops plasma-damaging complement factors from activating. This was determined by comparing two distinct populations, the Mediterranean and Northern Ireland, and it was discovered that there were no appreciable differences between them in terms of the healthier cardiometabolic characteristics that the first population displayed as a result of the higher plasma concentration of lutein and other antioxidants [20]. A relationship between vitamin A and subclinical atherosclerosis was discovered by assessing circulating retinol-binding protein 4 (RBP4), transthyretin (TTR), and the carotid intima-media thickness test (CIMT). Another study states that RBP4 is associated with insulin resistance, diabetes, and atherosclerosis, with retinol being inversely connected to CIMT. Dietary vitamin A showed a significant protective impact against preclinical atherosclerosis, and RBP4 levelss were connected to a specific involvement in atherogenesis [21].

Both studies, ATBC (Alpha-Tocopherol Beta-Carotene Cancer Prevention) and CARET (Beta-Carotene and Retinol Efficacy Trial) assessed the efficacy of beta-carotene in cancer prevention in smokers and included individuals with occupational asbestos exposure [22, 23]; which show no benefit from beta-carotene in terms of the incidence of CVD.

Retinol increases the production of tyrosine hydroxylase, the rate-limiting enzyme in the synthesis of catecholamines, via activating the nuclear retinoid receptors, according to another study [24]. In two successive, non-genomic pathways, Gelain *et al*. [24] show how retinol stimulates tyrosine hydroxylase. In patients with heart failure with a low left ventricular ejection fraction, blocking β-adrenoceptors lowers sympathetic nervous system activity and increases survival (LVEF). Another study looked into whether vitamin A status altered the relationship between using β-blockers and the risk of all-cause mortality [25]. It was shown that β-blocker therapy was related with better survival in patients with suspected coronary heart disease (CHD), particularly in those with high serum vitamin A levels. Excessive intake of preformed vitamin A, compared to its precursors, has been linked to teratogenicity in human and animal studies [26]. In women taking over 10,000 IUs of preformed vitamin A per day from supplements, it is estimated that 1 of 57 babies is born with a secondary congenital disability [27]. The patient may develop xerosis of the skin, and mucosa due to systemic use. Alopecia, anorexia, pruritus, mucous membrane dryness, muscle and bone pain, and hyperlipidemia are all symptoms of chronic poisoning [28].

### 2.2 Vitamin B

B vitamins are a group of eight water-soluble chemicals with chemically different structures that play a crucial role in cell metabolism as coenzymes in several anabolic and catabolic enzymatic activities [29]. Individual B supplements can be used separately because each one has specific properties, such as being a cofactor for essential metabolic processes or a precursor in the same area [30].

#### 2.2.1 Vitamin B1 or Thiamine (Aneurin)

Vitamin B1 or Thiamine is an essential component of glucose metabolism. It protects against atherosclerosis by counteracting the damaging effects of high glucose doses on the vascular wall, including chronic inflammation via lipid peroxidation, injury, and infection, all of which increase the risk of dyslipidemia [31]. The intravenous thiamine injection improved cardiac and hemodynamic functions and decreased vascular resistance. Endothelium-dependent vasodilation improved in individuals with hyperglycemia, leading to the recommendation that thiamine should be given regularly to decrease atherogenesis and increase endothelial function [32]. Another study evaluated endothelium-dependent vasodilation in three patient groups: healthy, impaired glucose tolerance, and non-insulin-dependent diabetes, using duplex ultrasonography to quantify brachial artery vasoactivity. It came to the conclusion that thiamine had outstanding effects on endothelial function and the onset of subclinical atherosclerotic disease in hyperglycemic patients predisposed to accelerated atherosclerosis [32]. In other studies, supplementing with vitamin B1 has been shown to significantly reduce vascular inflammation, with negative associations between high levels of thiamine and low-density lipoprotein cholesterol (LDLc), correspondingly, triglycerides. As a result, continuous vitamin B1 administration may be thought to slow the progression of atherosclerosis [33]. Vitamin B1 can be used as both a marker and a therapeutic agent, and it is thought to play a role in the development and prognosis of atherosclerotic vascular pathology [34]. Thiamine and its derivatives have been shown in both *in vitro* and animal models to be effective antioxidants that can improve endothelial function in both euglycemic and hyperglycemic conditions. Furthermore, thiamine administration was shown to lower urine microalbumin in both rat experiments and a randomised, double-blind placebo-controlled human pilot study [35, 36]. These findings suggest that thiamine has a significant cardioprotective effect, as urine microalbuminuria, an early predictor of diabetic DM nephropathy, is linked to an increased risk of CVD (cardiovascular disease) in both diabetics and non-diabetics [37]. Many studies have shown the prevalence of thiamine deficiency in patients with HF (3–91%) [38]. According to DiNicolantonio *et al*. [39] meta-analysis of two trials, thiamine supplementation significantly enhanced the net change in left LVEF (3.28%; 95% CI: 0.64% to 5.93%). Patients in the intensive care unit with cardiac and pulmonary insufficiency (N = 6) were given thiamine injections in progressive doses by Freye *et al*. [40] where it was found that patients receiving thiamine had a blood pressure increase of at least 20 mm Hg, and a moderate elevation of central venous pressure of 3 mm Hg, without a rise in heart rate, compared to the control group. This demonstrates that thiamine may act in these HF patients as a mild peripheral vasodilator, lowering afterload and increasing cardiac output. These studies show that thiamine supplementation may help patients with systolic HF improve their LVEF even though it is typically not indicated for patients with systolic HF [40].

It is scientifically conceivable to think that proper thiamine supplementation in persons with diabetes could greatly affect metabolic compensation and subsequently the emergence of vascular issues because of its function as an enzyme cofactor in glucose metabolism. It may also impact early phases of poor glucose tolerance, such as metabolic syndrome components. In addition to diabetes, data on surrogate markers of endothelial dysfunction (ED) and CVD suggest that thiamine may be helpful in a more extensive range of disorders. While experimental research has mainly shown that thiamine supplementation/therapy has a positive effect, clinical investigations of sufficient size and length concentrating on the impact of thiamine supplementation/therapy on hard endpoints are currently lacking. Furthermore, it is presently unknown which processes contribute the most to thiamine deficiency. Based on the facts presented, boosting plasma levels alone may not be the best approach because intracellular TDP levels are not a simple reflection of their precursor’s plasma levels. Additional experimental research into the molecular processes of thiamine shortage in diabetes is required before providing a definitive response to the diabetic community [41]. It is unlikely that thiamine intake from food sources alone would reach a hazardous level. When nutrient intake is exceedingly high, the body will absorb less of the nutrient and excrete any extra through urine. There is no known toxic level of thiamin [42]. A study investigating the safety of intravenous thiamine for Wernicke’s encephalopathy reported a total of 12 adverse reactions (1.1%). Therefore, it was concluded that thiamine hydrochloride might be administered intravenously without undue concern [43].

#### 2.2.2 Vitamin B2 or Riboflavin

Foods naturally contain vitamin B2, also known as riboflavin, and is also added to foods and sold as a supplement. Small amounts of riboflavin can be produced by gut bacteria, but not enough to satisfy dietary needs. It is a water-soluble vitamin that exists in two forms, as flavin adenine mononucleotide (FMN) and flavin adenine dinucleotide, both of which function as cofactors (FAD). It is typically present in its cofactor forms, linked to proteins, in a wide variety of meals, primarily meat and dairy. According to a study, vitamin B2 in its FAD coenzyme form plays a significant role in the glutathione redox cycle [44]. Riboflavin deficiency can lead to an increase in oxidized glutathione which results in cellular damage leading to an increase in lipid peroxidation, which can directly impact atherogenic pathways [44]. Riboflavin status influences lipid peroxide generation, implying that oxidative stress is intimately linked to vitamin B2 [45]. These findings show that riboflavin plays a vital role as an antioxidant in the human body’s defense system. Reduced consumption can influence the atherosclerotic process by disrupting the oxidant-antioxidant ratio [46]. It has been established that dihydroriboflavin, which is produced from riboflavin by NADPH-dependent flavin reductase, is an efficient reducing agent for heme proteins containing ferric iron and, therefore, a potential antioxidant. Interesting research reveals that riboflavin may prevent tissue damage brought on by ischemia-reperfusion. This protection is most likely provided by dihydro riboflavin, which most likely works with flavin reductase to eliminate oxidised heme proteins [47, 48, 49].

The role of plasma homocysteine as a graded risk factor for CVD has received much attention in recent years [50, 51]. Homocysteine is a thiol-containing amino acid that is formed when the essential amino acid methionine is broken down. It is metabolised by two main pathways: remethylation to methionine, which is reliant on folate, vitamin B-12, and riboflavin; and transsulfuration, which is reliant on vitamin B-6. Evidence for riboflavin’s function in homocysteine homeostasis comes from a report of elevated homocysteine levels in the skin of riboflavin-deficient rats [52]. In healthy people, particularly in those homozygous for the prevalent 677CT mutation, riboflavin status was found to be a regulator of plasma homocysteine levels [53]. The Framingham Offspring Cohort demonstrated that riboflavin intake influenced the total plasma homocysteine equally in both men and women [54]. Another study found a folate-riboflavin interaction that is unrelated to genotype in influencing plasma homocysteine [55].

Riboflavin levels in food and supplements have not been found to be toxic. The gut can only absorb so much riboflavin at one time, and any excess is quickly excreted in the urine [56]. As a result, there is no tolerable upper intake level for riboflavin.

#### 2.2.3 Vitamin B3 or Niacin

Niacin, a water-soluble vitamin, is essential for the metabolism and energy of cells. It can be found in a variety of foods, such as dairy, grains, and meat. Niacin is a precursor of the compound’s nicotinamide adenosine dinucleotide (NAD+) and nicotinamide adenosine dinucleotide phosphate (NADP+), which are crucial for the metabolic processes of glycolysis, the tricarboxylic acid cycle, the respiratory chain, and other processes requiring electron transport [57]. Niacin deficiency causes oxidative stress via affecting the cell’s redox equilibrium, which explains the lower levels of antioxidant enzymes, and by interfering with glutathione regeneration, which directly affects deactivating peroxides as a stress response [58]. Clinical trials have shown that niacin interferes with atherogenic process by raising plasma high-density lipoprotein cholesterol (HDLc) while decreasing proatherogenic lipids and lipoproteins, HDLc is an independent risk factor for CVD, with lower levels found in healthy women and men [59]. NA also inhibits ED by enhancing the arterial wall’s responsiveness to proinflammatory and prothrombotic stimuli, as well as its effect on endothelial cell-released inhibitors of vasorelaxation [60].

After a 15-year follow-up, the Coronary Drug Project, which evaluated the effectiveness and safety of five lipid-modifying medications, including niacin, discovered that niacin had a lower late mortality rate than placebo [61]. Multiple studies have examined the effect of niacin on the atherosclerotic plaque, with most of them involving statins or other CVD-related pharmaco-active drugs, yielding favorable results [62]. A trial group of 50 metabolic syndrome patients with no baseline CVD was randomized to receive a placebo or 1g extended-release niacin; the results concluded that endothelial function improved by 22% in the niacin group compared to the control. Thus, extended-release niacin therapy is likely to improve metabolic parameters by raising HDL-c, lowering triglycerides and atherosclerotic plaques [63].

Randomized Control Trials (RCTs) evaluating the possible benefit of niacin in association with of statin medication Simvastatin, did not show a similar benefit. In comparison to the placebo group, a sizable proportion of patients in the niacin group developed side effects such myopathy, rhabdomyolysis, and abnormal liver function tests [64]. In the Heart Protection Study 2 trial, the niacin group had a similar rate of side effects [65]. In his recently published a meta-analysis Jenkins *et al*. [66] found that using slow-release niacin did not improve CVD outcomes in individuals on statin medication but does raise the risk of all-cause mortality. Another study reported moderate-quality evidence of niacin significantly increasing the number of participants terminating treatment due to side effects, including flushing, pruritus, rash, and gastrointestinal symptoms, as well as severe new-onset diabetes [67].

#### 2.2.4 Vitamin B5 or Pantothenic Acid

Pantothenic acid (PA) is a coenzyme A (CoA) precursor that is required for metabolic activities like the Krebs cycle, fatty acid metabolism, and B-oxidation [68]. C-reactive protein (CRP), a circulating inflammatory marker, can convert oxidised LDLc into foam cells and serve as a long-lasting biomarker for low-grade inflammation, which is especially common in preclinical atherosclerosis [68]. Several studies have found a link between PA and CRP levels, as well as an inverse relationship between LDLc and triglyceride levels, but the mechanisms are still unknown [69, 70]. Pantethine is a nutrient that is produced physiologically in the human body from PA and has been taken as a dietary supplement since 1992. Studies have shown that it significantly lowers serum concentrations of LDLc and total cholesterol [71].

Plasma lipid concentrations are essential in the development and progression of atherosclerosis. Vitamin B5, as a precursor of CoA, has a positive effect on triglyceride synthesis and lipoprotein metabolism, which contributes to the onset and progression of atherosclerosis [34]. Compared to placebo, a 16-week treatment with pantethine in women with underlying CVD, lowered LDLc and total cholesterol, with a substantial reduction in the lipid profile. In addition, women experienced a better decline in non-HDLc levels compared to men, who had higher LDLc fraction findings [72].

#### 2.2.5 Vitamin B6 or Pyridoxal Phosphate (PLP)

Vitamin B6 comprises a group of six water-soluble chemical compounds which includes pyridoxal (PL), pyridoxamine (PM), pyridoxine (PN), and their $5^{\prime }$-phosphates [73]. About 160 bodily functions require the cofactor pyridoxal phosphate (PLP), which is the active form. It takes part in the transformation of lipids, nucleic acids, amino acids, and carbohydrates, as well as the breakdown of glycogen. The main metabolic processes that vitamin B6 regulates include the breakdown of tryptophan [74], sphingosine phosphate [75], and the activity of the transcription factor NF-B. By modulating the activity of inflammasomes and particularly its component, the NLRP3 sensory protein, it has the effect of lowering inflammation in the body [76]. The effect of vitamin B6 in CVDs and in decreasing blood pressure is one of the benefits of the vitamin that is frequently highlighted. Humans with high-risk atherosclerosis, strokes, and thrombosis also have low plasma levels of PLP [76, 77, 78]. A study on the effects of B6 on a sizable population of Koreans was undertaken by Jeon and Park. They found that higher vitamin B6 intake reduced the chance of developing CVDs in males, but there was no correlation in women [79]. Page carried out research on the effects of PLP concentration on post-menopausal women [80]. The conclusion was that the risk of myocardial infarction is inversely related to the concentration in plasma.

**Fig. 1.**
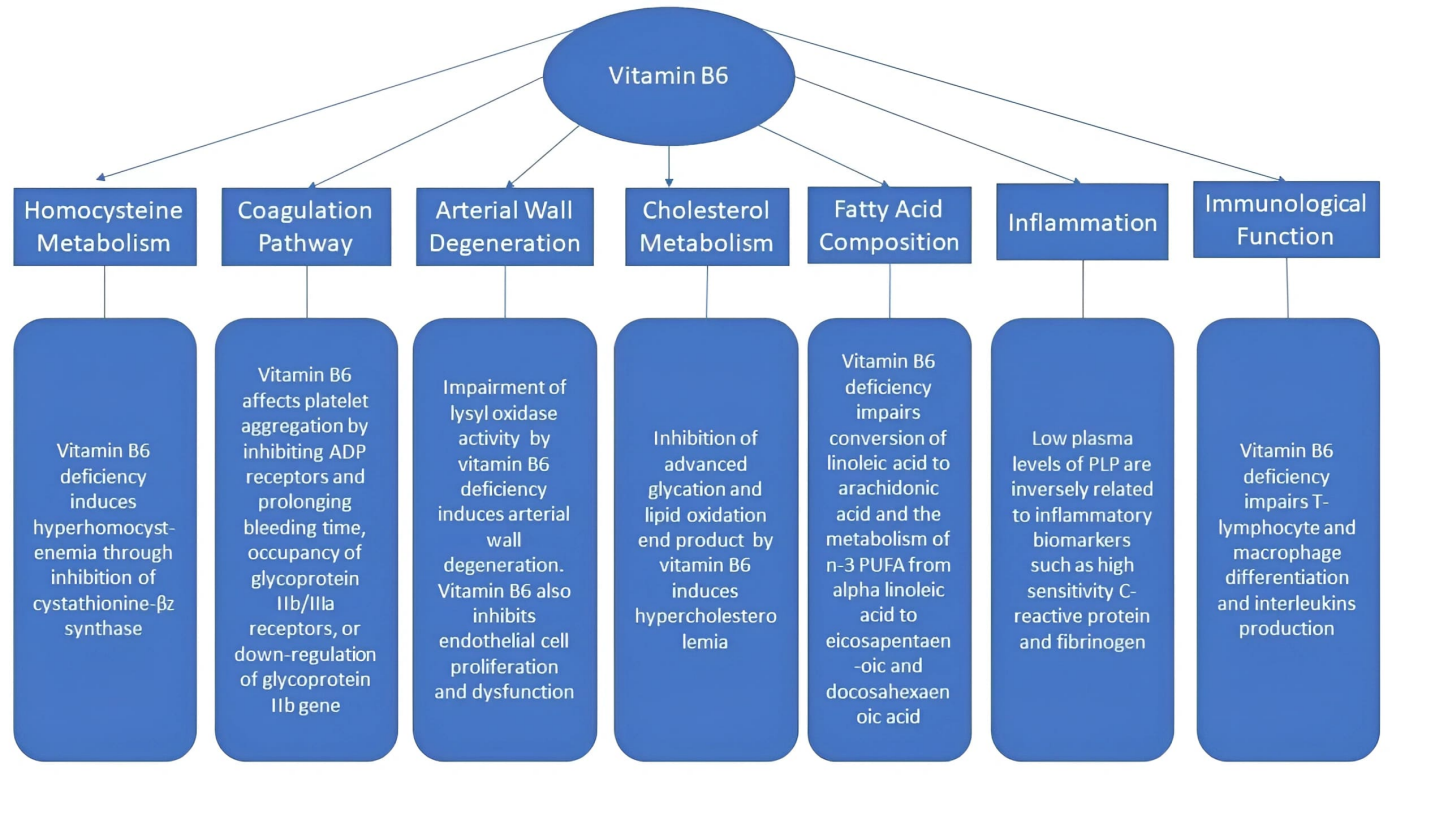
**Mechanisms of the atherogenic effects linked to vitamin B6**.

Recent epidemiologic studies have found a link between low vitamin B6 level and an increased risk of cardiovascular disease, albeit the precise mechanism is still unknown despite several potential explanations. The fact that vitamin B6 is a coenzyme for numerous processes suggests that it may have a cardioprotective impact. PLP is a coenzyme that is required for numerous metabolic processes, and it has also been linked to inflammation [81, 82], immunological function [82] thrombosis [83, 84], all of which are crucial factors across all phases of the atherosclerotic process. Fig. 1 summarises the proposed candidate processes for atherogenesis linked to vitamin B6.

#### 2.2.6 Vitamin B7 or Biotin

Biotin is a vitamin found in meat, eggs, grains, and vegetables in their natural state. It participates in gluconeogenesis, the metabolism of fatty acids, and certain carboxylation processes as a cofactor [85]. There are currently no studies to prove that biotin deficiency affects atherogenesis or prevents atherosclerosis [86].

#### 2.2.7 Vitamin B9 or Folic Acid

Homocysteine is positively associated with an increased risk for CVD. Folic acid may help to reduce the risk of CVD by lowering homocysteine levels in the blood. Folic acid and endothelial nitric oxide synthase (eNOS) worked together to increase endothelial function over the course of chronic treatment [87]. By lowering plasma homocysteine levels or by directly interacting with eNOS to prevent the production of reactive oxygen species, vitamin B9 may be a useful therapy option for minimising inflammation in preclinical atherosclerosis. Folic acid lowers homocysteine levels and influences eNOS in several CV predictive parameters such as blood pressure, arterial stiffness, endothelial function, and prothrombotic activity following acute or chronic treatment [88].

Homocysteine has been proposed as a modifiable risk factor for CVD in many studies. Homocysteine induces oxidative stress, destroys endothelium, and increases thrombogenicity in animal studies [89, 90, 91]. Homocysteine levels and cardiovascular risk have been linked in epidemiologic research progressively and independently [51, 92, 93, 94]. This finding is significant because homocysteine levels can be easily managed with safe and inexpensive medication.

A four-week experiment on otherwise healthy cigarette smokers showed that treatment with folic acid dramatically lowered homocysteine and fibrinogen levels compared to placebo [95]. The China Stroke Primary Prevention compared adults with hypertension but no prior history of MI or stroke to those taking enalapril with folic acid [96]. After a median follow-up of 4.5 years, the incidence of ischemic stroke and composite cardiovascular events was significantly lower in the group receiving enalapril with folic acid. Patients with low baseline folate levels saw more significant benefits.

Although folic acid (pteroylglutamic acid) taken orally is typically not hazardous to healthy people, it can cause neurological injury if they have untreated pernicious anaemia. Drug-treated epileptic patients should use caution when taking the vitamin because seizure control may be impaired. Folic acid supplements reduce intestinal zinc absorption in people and animals, according to some studies while other research contradicts this. The weight of evidence indicates that daily folic acid supplementation of 5–15 mg has no notable adverse effects [97].

#### 2.2.8 Vitamin B12 or Cobalamin

The water-soluble vitamin cobalamin, which is required for cell metabolism, can be found in diet, notably in animal products [98]. Numerous research looked into the relationship between cardiovascular health and vitamin B12 intake. Vitamin B12 levels have also been linked to unfavourable blood lipid profiles in people with type 2 diabetes mellitus, particularly with regard to triglycerides and the cholesterol/HDL ratio through inhibition of carnitine palmitoyl transferase, the rate-limiting enzyme of fatty acid oxidation [99]. By limiting homocysteine conversion to methionine, vitamin B12 deficiency increased homocysteine, a risk factor for CVD [100]. Low B12 vitamin levels are thought to raise cardiovascular risk, partly through direct effects [101]. Hyperhomocysteinemia caused by dietary deficit is mild but leads to higher risk of atherogenesis through endoplasmic reticulum stress, pro-inflammatory response, and oxidative stress are linked to a When 158 healthy siblings of patients with premature atherothrombotic disease received cobalamin therapy to lower homocysteinemia, the incidence of atherosclerotic events was lower than in the placebo group [102]. In a different study, it was discovered that males with higher homocysteine levels have a higher risk of having an atherothrombotic process [103]. The “B-Vitamin Atherosclerosis Interventional Trial” (BVAIT) investigated whether homocysteine is a marker or the root cause of atherosclerotic vascular pathology in light of the association between high homocysteine levels and an increased risk of CVD (cardiovascular disease) and produced a number of encouraging results. In individuals without a history of CVD, high vitamin B dosages (folate 5 mg, vitamin B12 0.4 mg, and vitamin B6 50 mg) reduced the onset of subclinical atherosclerosis [104].

However, a meta-analysis assessing the relationship between vitamin B supplementation and cardiovascular outcomes found that it had little to no impact on reducing CVD events (RR, 0.98; 95% confidence interval [CI]: 0.93–1.03; *p* = 0.37), total mortality (RR, 1.01; 95% CI: 0.97–1.05; *p* = 0.77), cardiac death (RR, 0.96; 95% CI: 0.90–1.02; *p* = 0.21), MI (RR, 0.99; 95% CI: 0.93–1.06; *p* = 0.82), or stroke (RR, 0.94; 95% CI: 0.85–1.03; *p* = 0.18). Although in some patient subgroups, initial CVD prevention appeared to be more beneficial than secondary prevention when vitamin B intake was increased [105]. In general, studies have not revealed any effects of specific B vitamin supplements on the development of subclinical atherosclerosis in those without baseline CVD. Despite the paucity of research on the clinical effects of excess vitamin B12 caused by multiple oral doses of cyanocobalamin, a case of a woman was reported who received multiple daily doses of 1 mg of cyanocobalamin for severe pernicious anaemia was reported. She experienced acne, palpitations, anxiety, akathisia, facial ruddiness, headaches, and insomnia, which subsided two weeks after the drug trial was stopped [106].

### 2.3 Vitamin C 

Vitamin C, also known as ascorbic acid, serves as an important micronutrient and antioxidant for the body. It functions as a reducing agent as it acts as an electron donor while itself being oxidized to a radical, thereby helping in mediating reactive oxygen species-induced damage, as well as regenerating other antioxidants [107]. It has both enzymatic and non-enzymatic functions, however, in the context of cardiovascular health, vitamin C has shown to have positive impacts on reducing oxidative stress and damage, blood pressure, and improving endothelial function among other risk factors of atherosclerosis [108, 109, 110, 111].

Various types of studies have highlighted the benefit of vitamin C on cardiovascular health, attributing its positive impacts to its antioxidant capabilities. One review established that supplementation with vitamin C reduced systolic and diastolic blood pressures [112], and another study suggested that vitamin C has a protective effect on arterial stiffness [113], both associated factors for atherosclerosis. A review of RCTs determined the effect of ascorbic acid supplementation on endothelial function. The authors found that an improvement in endothelial function was seen in doses greater than 500 mg/d and a better effect was noted in those with a higher CVD risk [109]. Antioxidants like vitamin C have been found to contribute to endothelial function by regulating nitric oxide level of the endothelium, and impeding inflammation, lipid peroxidation, platelet oxidation, and LDL oxidation - factors that can worsen the status of ED [114]. In the European Prospective Investigation of Cancer (EPIC) - Norfolk study, plasma ascorbic acid’s relationship with mortality was examined in 19,496 men and women. An inverse relationship existed between ascorbic acid and cardiovascular risk factors. Plasma ascorbic acid concentration was also found to be inversely related to ischemic heart disease (IHD) and mortality due to CVD [115]. A meta-analysis of 16 prospective studies found a notable inverse relationship between dietary consumption and circulating vitamin C levels and the risk of stroke [116].

Vitamin C is generally tolerable at high dosages in healthy individuals. There is a possibility that high levels may lead to the formation of kidney stones, risk of iron overload, or even hemolysis in glucose-6-phosphate dehydrogenase deficient people [117]. There is very limited data on the negative impacts of vitamin C-only supplementation on cardiovascular health. One important study to be noted is the Iowa Women’s Health Study which examined how vitamin C would alter the mortality from CVD in postmenopausal diabetic women. A disturbing association was noted; supplementing high doses of vitamin C was associated with an increased risk of CVD mortality [118]. Other negative association of vitamin C in postmenopausal women has been noted while being co-supplemented with vitamin E, further explained later in the article in the ‘recommendations’ section.

To summarize, vitamin C is one of the most important vitamins required for cardiovascular health. Though few studies are highlighting the risks in postmenopausal women, a regular intake should be recommended to individuals at risk of any form of CVD.

### 2.4 Vitamin D

The group of secosteroids, Vitamin D, well known for its vital role in homeostasis of calcium and bone mineralization, falls under one of the most common nutritional deficiencies worldwide, prevalent in more than one billion people [119]. Even though the effects of this vitamin have been widely researched in various medical conditions and diseases, establishing a strong clinical relation between vitamin D and its influence on cardiovascular health has been difficult. Since the finding of vitamin D receptor (VDR), an intracellular receptor, in various cardiovascular tissues such as cardiomyocytes, endothelial cells, smooth muscle cells of the vasculature, and platelets, amongst others [120], the topic of vitamin D’s role in CVD has been of much debate. In addition to the VDR, vitamin D–binding proteins in the blood circulation, whose concentration and binding affinity, if affected, has been stated to lead to a risk of vitamin D deficiency and other CVDs [121].

Evidence from existing literature documents various roles of vitamin D which have been postulated in modulating CVD, such as atherosclerosis, blood pressure, IHD, and cardiac failure [120, 122, 123]. Since VDRs are also found on vascular endothelial cells, studies have revealed possible vitamin D effects linked to endothelial function. In a study by Zhang *et al*. [124], endothelial function was assessed by measuring and comparing brachial artery flow-mediated dilation (BAFMD), sE-selectin and soluble vascular cell adhesion molecule-1 in 117 non-dialysis chronic kidney disease (CKD) patients. The results concluded that BAFMD was lower in patients with low-to-no vitamin D levels than in individuals with normal vitamin D levels. The remaining findings also showed associations and correlations that indicated low-to-deficient vitamin D levels with ED [124]. With the relation between vitamin D deficiency and ED, and the fact that ED also correlates to an increased risk in CVD mainly by contributing to hypertension and atherosclerosis [122, 125], it can be assumed that there is indirect causation of vitamin D deficiency in CVD. Many studies have also shown an inverse relationship between plasma 25(OH)D levels and hypertension, one even concluding that vitamin D can be used as an ‘adjuvant therapy for patients with grades I–II essential hypertension’ [126, 127]. The protective nature of vitamin D against myocardial infarction (MI) and stroke has been hypothesized and studied, mainly displaying results in favor of the hypothesis. In a review by Muscogiuri [123] in which the effects of vitamin D on CVD, mainly MI and stroke was explored, studies confirmed that vitamin D might protect against these CVDs, in contrast to a few that proposed the opposite. However, it was made sure that hypovitaminosis D promotes atheroma formation and increases the risk of vessel wall calcification, factors both which contribute heavily to major CVDs [128].

Large-scale randomized trials have produced results that do not favor the hypothesis of vitamin D supplementation providing any benefit against CVD. For example, VITAL, a randomized, double-blind, placebo-controlled trial to examine the benefits and risks of vitamin $D_{3}$, supplemented a dosage of 2000 IU/day to over 25,800 adults across the U.S. After a median 5.3-year intervention, the study concluded that daily high dosage vitamin D did not affect the incidence of any cardiovascular events, major or minor [128]. Another randomized clinical trial exploring the effect of monthly high dosage supplementation also yielded similar results [129]. A Mendelian randomization study tested the association of genetically reduced plasma 25(OH)D with the increased risk of IHD and MI and found no evidence of such association [130]. In addition, recent randomized controlled trials have had results indicating no benefit of vitamin D on the management of hypertension [131, 132]. With many studies highlighting hypovitaminosis D and its correlation with CVD risk, the exact nature of the relationship between vitamin D and cardiac vasculature remains disputable.

### 2.5 Vitamin E

Vitamin E is an essential, lipid-soluble, non-enzymatic antioxidant, which exists in 2 primary forms, tocopherols and tocotrienols, each having four subtypes (a-, b-, g-, and d-) [133]. Its importance mainly lies with the anti-inflammatory and antioxidant properties, as seen in numerous *in vitro* and animal studies. Its role in lipid peroxidation has mainly brought forward the idea of its potential cardioprotective effects.

Studies have usually shown an inverse association between plasma concentrations of antioxidants and CVD [134, 135]. A possible mode of action of vitamin E that likely contributes to reduced risk of CVD is through the antioxidant protection of LDL [136]. Oxidized LDL attracts circulating monocytes and inhibits macrophage movement in the intima. Oxidatively modified LDL uptake via scavenger receptors of macrophages, therefore, then leads to cholesterol-laden foam cells and fatty-streak formation - causing atherosclerosis. Another way of preventing formations and/or progression of the fatty streak is via inhibiting platelet adherence and aggregation, which vitamin E is believed to carry out [137]. In oxidative stress, a decrease in d-a-tocopherol levels from smooth muscle cells results in the growth and proliferation of these cells, which contributes to atherosclerosis [136].

Patients undergoing hemodialysis treatment for end-stage renal disease have been found to have an increased risk of cardiovascular events [138]. A study by Mune *et al*. [139] investigated the vitality of vitamin E supplementation on HDL and endothelial function in end-stage renal disease (ESRD) patients undergoing hemodialysis. The study showed promising positive results and concluded that the supplementation significantly improved HDL function of cholesterol efflux capacity and endothelial function [139]. A daily supplement of 400–800 IU is recommended in secondary prevention of cardiovascular events in maintenance hemodialysis patients [140]. A 30-year prospective cohort analysis analyzed the effect of serum α-tocopherol on long-term overall and cause-specific mortality. The results defined that increased baseline serum α-tocopherol levels were linked with lower risk of overall and all significant causes of mortality [141].

Studies have highlighted that there are no effects and/or adverse effects on the cardiovascular system due to vitamin E supplementation. The Physicians’ Health Study II randomized trial explored the effect of long-term supplementation of vitamin E and C on the risk of CV events in over 14,600 men. After ten years, the study concluded that vitamin E or vitamin C had no significant effects on the primary endpoint of major cardiovascular events, individual cardiovascular events (including MI and total stroke), cardiovascular mortality, and adverse effects such as hematuria, easy bruising, and epistaxis. However, it is to be noted that a significant association was seen between vitamin E and increased risk of hemorrhagic stroke (HR, 1.74; 95% CI, 1.04–2.91; *p* = 0.036) [142]. In a trial in 28,519 male cigarette smokers, a 50% higher risk of subarachnoid hemorrhage with 50 mg per day of α-tocopherol supplementation, while no effect was seen on the risk of intracerebral hemorrhage [143]. In a Mendelian randomization study by Wang* et al*. [144], higher vitamin E levels were related with increased possibilities of CAD and MI. Furthermore, vitamin E levels were also appreciably linked with CAD risk factors, including LDL-cholesterol, total cholesterol, and triglycerides [144]. The HOPE-TOO study, which was an extension of the al Heart Outcomes Prevention Evaluation [HOPE] trial, monitored the long-term supplementation effects of vitamin E and found a somewhat concerning vitamin E effect, as it stated that though there was no significant effect on major CVD events and total mortality, increased HF rates were noted in patients supplemented with vitamin E [145].

It is possible that vitamin E only offers benefits to cardiovascular health in certain patient groups such as those under high oxidative stress, however, more insight regarding the relation of its antioxidant nature with reducing CVD risk is required.

### 2.6 Vitamin K

**Fig. 2.**
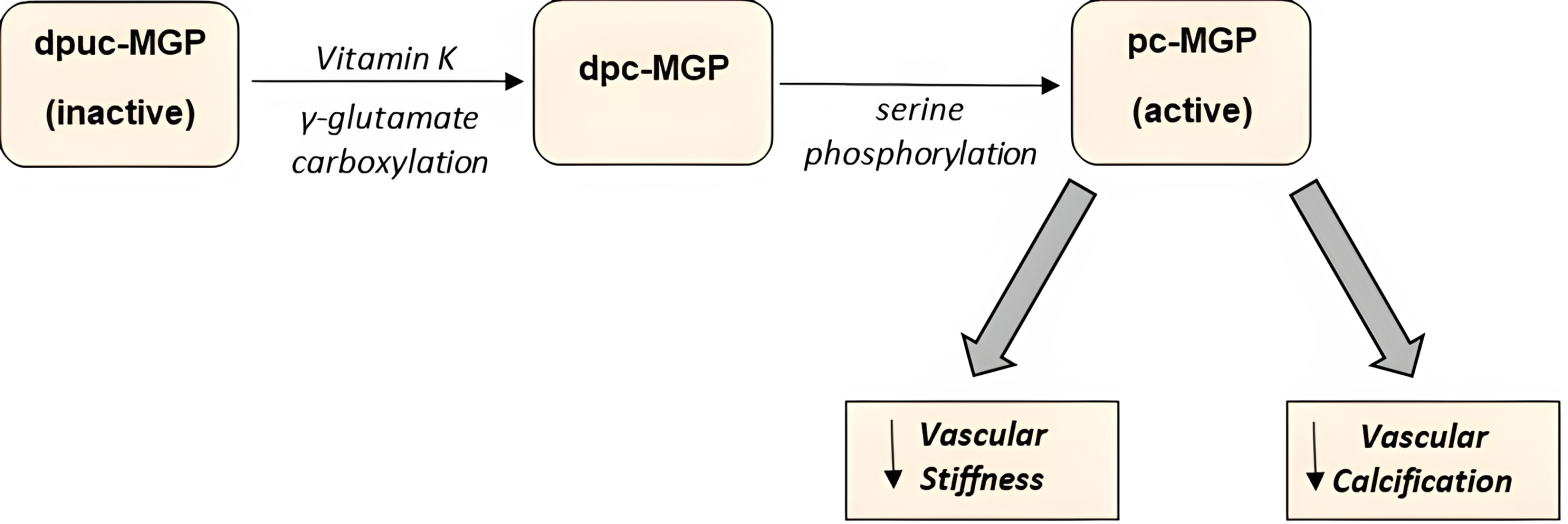
**Two-step activation of the Matrix Gla Protein**. dpuc, dephosphorylated uncarboxylated; dpc, dephosphorylated carboxylated; pc, phosphorylated carboxylated.

Vitamin K, which occurs in two forms, vitamin K-1 (phylloquinone) and vitamin K-2 (menaquinones (MKs) [146], is well known for its essential role in hemostasis, where it serves as a cofactor for coagulative and anticoagulative factors’ synthesis. It plays a vital role in the management of CVD besides the coagulative pathway. It is responsible for the first part of a two-step activation of Matrix Gla Protein (MGP) (as shown in Fig. 2), a vitamin K-dependent protein (VKDP) that is required for impeding vascular calcification (VC); a characteristic marker of atherosclerosis, especially in coronary arteries [146]. In fact, in patients diagnosed with CKD, for whom the most common cause of death is CVD, mainly due to the fatal progression of VC, vitamin K supplementation has been explored to decrease CVD-specific mortality [147, 148]. MGP is released by chondrocytes and vascular smooth muscle cells (VSMCs). A laboratory study on mouse models found that of MGP in arteries rescues only the arterial mineralization aspect of the MGP deficient mice, whereas its expression in osteoblasts inhibits bone mineralization [149]. This means that increased systemic levels of active MGP will not affect vascular calcification, indicating the importance of its synthesis via VSMCs and hence adequate vitamin K levels. To identify the vitamin deficiency status, the levels of desphospho-uncarboxylated MGP (dp-ucMGP) or other VKDPs serve as serum markers [150].

Several studies have backed up the positive role of vitamin K in reducing the risk of CVD. A population-based study, the Rotterdam Study, examined the effects of the intake of both forms of vitamin K primarily on aortic calcification and coronary heart disease (CHD) in 4807 participants. The results showed that though phylloquinone intake had no association with any CVD events, menaquinone intake was significantly associated with the decreased risk of incident-CHD (RR = 0.59), CHD mortality (RR = 0.43), and all-cause mortality (RR = 0.74). Additionally, with severe arterial calcification, a strong negative correlation with menaquinone intake was seen. This study concluded that menaquinone-rich diets might help prevent CHD, likely by mediating arterial calcification inhibition [151]. Zwakenberg *et al*. [152] reported that vitamin K intake had no association with CVD but noted the possibility of higher intake of long-chain menaquinones associated with a reduced risk of CHD mortality. Another prospective cohort analysis aimed to evaluate the association of the two forms of vitamin K with cardiovascular mortality concluded that both forms likely have a protective role in cardiovascular health [153]. Supplementation of phylloquinone was also shown to slow the progression of VC of the coronary artery in older adults who had varying forms of pre-existing calcification [154]. A high dose of vitamin K-2 intake was found to protect against CHD, associating this protective effect to the higher subtypes, mainly MK-7, MK-8, and MK-9 [155].

As all vitamins have mixed effects shown by various studies, vitamin K is no different. One study examining the association between forms of vitamin K’s effect on reduced stroke risk did not find any association between phylloquinone and menaquinones’ intake with stroke [156]. Another trial monitored whether supplementation of the vitamin K-2 would affect vascular stiffness in patients with CKD by primarily measuring the carotid-femoral pulse wave velocity and secondarily the blood pressure, abdominal aortic calcification, physical function, and serum markers of vascular health. The results indicated that K2 supplementation had no impact on vascular stiffness or calcification [150]. A randomized controlled trial assessing MK-7 supplementation on VC in type 2 diabetic patients showed increased active VC on 18F-NaF Positron emission tomography (PET) scan [157]. However, the fact that there was a lack of patients with vitamin K deficiency in the patients’ group could have influenced the results.

The effect of vitamin K supplementation does show a potential role in lowering the risk of CVD by reducing vascular stiffness and calcification. With few large-scale randomized controlled trials to date, more research and in-depth analysis is required to explore the detailed impact of vitamin K’s effects on reducing CVD risk.

## 3. Supplementation of Vitamins with Other Vitamins or Compounds

Vitamins C and E: Co-supplementation of these two antioxidant vitamins has been proposed to be beneficial due to their synergistic activity. It was reported that vitamin E acts as the primary antioxidant and thereby produces a radical, which is reduced and regenerated by vitamin C, producing a vitamin C radical. The vitamin C radical, in turn, is regenerated via the NADH-oxidation system [158]. A clinical trial by Salonen *et al*. [159] studied the efficacy of co-supplementation of the antioxidant vitamins E and C on the progression of carotid atherosclerosis in smoking and nonsmoking men and postmenopausal women. The authors concluded that with the proper doses of both vitamin E and slow-release vitamin C, the progression of common carotid atherosclerosis in men could be retarded [159]. A case-control study to assess the oxidative stress management in CVD by antioxidant vitamins concluded results that favored the supplementation of the stated combination [160]. However, this form of co-supplementation is not advised for postmenopausal women, as indicated in The Women’s Angiographic Vitamin and Estrogen (WAVE) Trial, where those taking high doses of vitamins C and E were found to have an increase in cardiovascular mortality [161].

Vitamin D and K: Vitamin D and K, both fat-soluble vitamins, have been shown to have a collaborative effect on human health. Vitamin D promotes the synthesis of VKDPs, which play an imperative role in cardiovascular health. A review by van Ballegooijen *et al*. [162] concluded that the appropriate concentrations of vitamin D and K supplemented together will be beneficial for reducing the risk of CVD, but a simultaneous intake of calcium can be harmful as it may likely contribute to soft tissue calcification and CVD. A prospective cohort study investigated the relation between vitamin D and K serum level markers and cardiovascular health markers such as LV systolic function and cardiac structure. In the group that had low levels of vitamin D and K, it was found that they had an increased left ventricular mass index and an increased risk of all-cause mortality [163].

Vitamin E and Magnesium: A recent meta-analysis evaluated the influence of magnesium and vitamin E co-supplementation on several CV risk factors in patients with metabolic disorders. A significant decrease in fasting plasma glucose, insulin, triglyceride, total cholesterol, and low-density lipoprotein was seen, while no noteworthy effect on the body weight, body mass index, and high-density lipoprotein in the patients was stated [164].

## 4. Recommendations

• It was addressed by Dr. M. C. Morris, and Dr. C. C. Tangney that many RCTs being conducted regarding vitamin supplementation indicate no positive or true results due to testing in the ‘wrong type’ of population. These trials should involve people with deficient status of vitamin levels rather than healthy individuals who supposedly have a relatively better intake [165].

• High doses of vitamin C in postmenopausal women are dangerous to cardiovascular health as stated earlier [118, 161]. Another study also found that increased vitamin C intake was associated with a greater risk of breast cancer in postmenopausal women [166]. Therefore, with the current lack of detailed insight regarding this, the use of vitamin supplements in this population should be explored and studied further. Until solid guidelines are formed, high dosage vitamin C supplementation should not be recommended to postmenopausal women, especially those suffering from one or more chronic diseases.

• Increased levels of homocysteine are an important modulator for the development of cardiovascular and peripheral arterial disease. Regulating adequate levels of vitamins involved in regulating this pathway can substantially reduce the risk of CVD and its associated co-morbidities. This can be done via supplementation with Vitamin B2, B6, B9, and B12.

• It’s also important to remember that taking supplements can have adverse effects that appear gradually. Numerous RCTs have documented both significant and minor negative effects. The ATBC and CARET studies focused on the potential benefits of beta-carotene supplementation in people with a high baseline risk of developing lung cancer, and both trials found that higher-risk participants receiving beta-carotene had a higher incidence of lung cancer [167]. In the vitamin E arm of PHS II, there was a documented rise in hemorrhagic stroke cases. Extended follow-up in the SELECT study revealed an elevated prostate cancer risk [168]. Rashes and mild bleeding incidents while taking multivitamins, gastrointestinal issues after taking beta-carotene, hypercarotenemia or yellowing of the skin, and gastrointestinal symptoms with selenium [167].

## 5. Conclusions

**Fig. 3.**
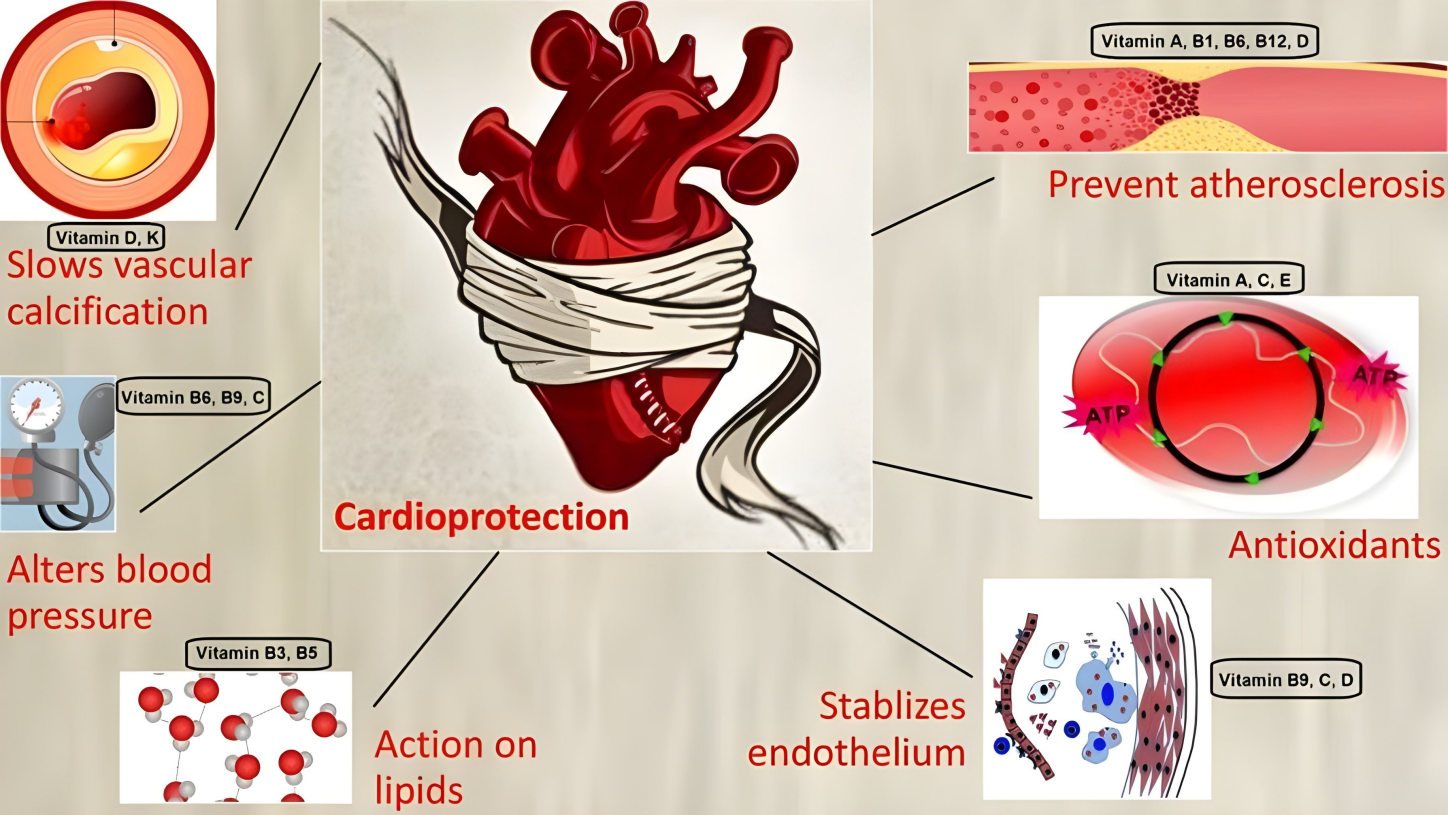
**Mechanism of action of vitamins offering cardioprotection**.

To summarize, in Table 1 (Ref. [17, 18, 20, 33, 34, 35, 53, 60, 64, 86, 88, 89, 90, 91, 102, 104, 105, 109, 110, 111, 112, 113, 114, 122, 124, 125, 128, 136, 137, 138, 139, 140, 151, 154]) the authors have briefly stated the promising outcomes of various vitamins’ supplementation on cardiovascular health. Fig. 3 outlines the mechanisms of action by which vitamins offer cardioprotection. In addition, although every vitamin has been found to decrease the chances of atherosclerosis in one way or the other, there are several studies also indicating no benefits and a few even hinting at its negative impacts, as highlighted in Table 2 (Ref. [23, 118, 142, 143, 144, 145, 148]). In Table 3 (Ref. [128, 150, 169, 170, 171, 172, 173, 174, 175, 176, 177, 178, 179]), we briefly outline some of the recent clinical trials that explored the relationship between vitamin supplementation and CVD.

**Table 1.** **A summary of the beneficial effects of vitamins on the cardiovascular system**.

Vitamin		Effect on cardiovascular system
A		Prevents atherosclerosis by
		∙ Inhibiting vascular smooth muscle cell proliferation, endothelial adhesion, and complement system [17]
		∙ Promotes fibrinolysis [18]
		∙ Antioxidant properties [20]
		∙ Modifies use of β-blockers and improves survival
B	B1	Slows progression of atherogenesis by
		∙ Decreases vascular resistance
		∙ Improves vasodilation
		∙ Decreasing vascular inflammation [33, 34, 35]
	B2	∙ Influences homocysteine levels which play an important role in atherogenesis [53]
	B3	∙ Raises Plasma HDLc [60]
		∙ Lowers TAGs [64]
	B5	∙ Reduces plasma LDLc and total cholesterol [34]
	B6	∙ Reduces atherosclerosis, blood pressure and risk of stroke [89, 90, 91]
	B7	No proven evidence [86]
	B9	∙ Lowers homocysteine levels
		∙ Influences endothelial nitric oxide synthase (eNOS) and alters blood pressure, arterial stiffness, endothelial function, and prothrombotic activity [88]
	B12	Prevents formation of atherosclerotic plaque by lowering homocysteine levels [102, 104, 105]
C		∙ Limits oxidative damage [110, 111]
		∙ Manages systolic and diastolic blood pressures [112, 113]
		∙ Enhances endothelial function [109, 114]
D		∙ Enhances endothelial function [122, 124, 125]
		∙ Obstructs atheroma formation [128]
		∙ Slows down vascular calcification [128]
E		∙ Decreased risk of CVD in ESRD patients undergoing hemodialysis [138, 139, 140]
		∙ Limits oxidative damage [136, 137]
K		∙ Slows down vascular calcification and stiffness via MGP [151, 154]

**Table 2.** **A summary of the harmful effects of vitamins on the cardiovascular system**.

Vitamin	Author	Type of study	Period of study	Patient population	Harmful effect
A	Omenn *et al*. [23]	RCT	1985–1998	1029 men and women with extensive histories of cigarette smoking	Rate of death due to cardiovascular causes was higher by 26 percent
C	Lee *et al*. [118]	Observational Study	1986–2000	1923 diabetic postmenopausal women	Increased risk of cardiovascular disease mortality
E	Sesso *et al*. [142]	RCT	1997–2007	14,600 men	Increased risk of hemorrhagic stroke
	Leppälä *et al*. [143]	RCT	1985–1993	28,519 male cigarette smokers	Increased risk of subarachnoid hemorrhage
	Wang *et al*. [144]	Mendelian Randomization	-	7781 individuals of European descent	Increased risk of coronary artery disease and myocardial infarction
	The HOPE and HOPE-TOO Trial Investigators [145]	RCT	1993–2003	7030 with vascular disease or diabetes mellitus	Increased rate of heart failure
C and E	Waters *et al*. [148]	RCT	1997–2002	423 postmenopausal women with varying degrees of coronary stenosis	Worsened mean lumen diameter and increased cardiovascular mortality

RCT, Randomized Controlled Trial.

**Table 3.** ** A review of clinical trials summarizing the effects of vitamins in cardiovascular health**.

Vitamin	Author	Period of study	Patient population	Control group	Primary outcomes	Secondary and/or other outcomes	Results
B1	Smithline *et al*. [169]	January 2008–June 2012	130 hospitalized patients with acute heart failure	60 participants	Effect of thiamine supplementation on dyspnea	Spirometry rate, type B natriuretic peptide, free fatty acids, hyperglycemia, length of stay in the hospital, 30-day rehospitalization, and death.	Standard thiamine treatment did not improve dyspnea, biomarkers, or other clinical parameters in patients with mild-moderate acute heart failure who did not have thiamine deficiency.
B2	RIBOGENE Trial [170]	February 2013–December 2017	243 participants	-	Blood Pressure	Erythrocyte Glutathione Reductase Activation Coefficient (EGRAC) [indicator of vitamin status], plasma homocysteine and red cell folate, vitamin B6 and vitamin B12.	Not Published.
	RAFA Trial [171]	June 2011–August 2019	2564 participants	-	Blood Pressure	Central blood pressure, pulse wave analysis, pulse wave velocity, red blood cell riboflavin, red blood cell folate, serum homocysteine, and plasma vitamin B6.	Not Published.
B3	Abott Study [172]	February 2010–May 2012	128 participants	-	Effectiveness of Niaspan (Niacin)	Evaluation of changes in non-HDL-C. lipids, LDL-C, total cholesterol, and triglycerides against base line values, frequency of flushing events and overall safety and tolerability of Niaspan.	Rise in mean HDL-c was noted.
B6	VITATOPS Trial Study Group [173]	November 1998–June 2009	8164 participants	4075 participants	Non-fatal stroke, non-fatal myocardial infarction, or death due to vascular causes	-	The use of B vitamins to prevent recurrent strokes is not supported by these results. The results of ongoing trials and individual patient data of the impact of B vitamins will have more statistical strength.
B9	SEARCH study collaborative group [174]	July 1998–May 2008	12,064 participants	6031 participants	Major Vascular Events (MVE)	MVEs individually in Years 1 and Later Years, MVEs in patients divided into 3 groups based on baseline LDL, MVEs in presence and absence of the other factorial therapy, Major Coronary Events, MI without death, MI with death or revascularization, and total strokes.	In this cohort, decreasing homocysteine with folic acid and vitamin B12 supplementation is not linked to a decline in cardiovascular events.
B12	G Bostom *et al*. [175]	May 2002–October 2011	4110 participants	-	Recurrent or de Novo Arteriosclerotic Cardiovascular Disease (CVD) Defined as the Occurrence of Non-fatal or Fatal Arteriosclerotic Outcomes Including Coronary Heart, Cerebrovascular, and Peripheral Vascular Disease Events	Renal Graft Failure, Mortality (All-cause), Fatal/Non-fatal Myocardial Infarction (MI), Fatal/Non-fatal Stroke, Resuscitated Sudden Death (RSD), CVD Death, Coronary Artery Revascularization), Lower Extremity Peripheral Arterial Disease (PAD), Carotid Endarterectomy or Angioplasty, Abdominal Aortic Aneurysm Repair, Renal Artery Revascularization.	Despite a considerable decrease in homocysteine levels, treatment with a high-dose folic acid, B6, and B12 multivitamin did not lower a composite cardiovascular disease outcome, all-cause mortality, or dialysis-dependent kidney failure in kidney transplant recipients.
D	Theiler-Schwetz *et al*. [176]	2011–2014	200 participants	100 participants	24-hour systolic ambulatory blood pressure	24-hour diastolic blood pressure, plasma aldosterone and renin concentration, and pulse wave velocity.	A marginal significant inverse relationship was noted between concentrations of 25(OH)D with 24-hour systolic BP. No significant treatment effect was noted on secondary outcome measures.
	VITAL/Manson *et al*. [128]	2010–2025	25,871	6474 (complete placebo)	Invasive cancer of any type and major cardiovascular events	Site-specific cancers, death from cancer, and additional cardiovascular events.	Vitamin D supplementation did not lower the incidence of invasive cancer or cardiovascular events in comparison to placebo.
	Angellotti* et al*. [177]	48 weeks	127 stable type-2 diabetic patients	61 participants	Disposition Index	Number of participants with change in glycemia. HbA1c, cardiovascular risk factors, effect on plasma concentrations of surrogate biomarkers of cholesterol.	One year of vitamin D3 supplementation of 4000 IU/day did not affect any of the outcome measures except that it led to an improvement of triglycerides among patients not on cholesterol medication.
K	The K4Kidneys Randomized Controlled Trial/Witham* et al*. [150]	12 months	159 participants with chronic kidney disease stage 3b or 4	79 participants	Difference in pulse wave velocity between-group at 12 months	Other markers of vascular health (e.g., augmentation index, abdominal aortic calcification, blood pressure, physical function, and blood markers of mineral metabolism and vascular health).	Vitamin K2 supplementation did not have any positive effect on vascular stiffness or other measures of vascular health.
	Mansour *et al*. [178]	March 2015–May 2016	60 participants	-	Reduction From Baseline in Carotid-femoral Pulse Wave Velocity at 8 Weeks	Change from baseline in central pressure and augmentation index by ambulatory hemodynamic measurement, and blood concentration of dephosphorylated-uncarboxylated Matrix Gla Protein (Dp-ucMGP) at 8 Weeks.	Vitamin K2 supplementation improved subclinical vitamin K deficiency and arterial stiffness in renal transplant recipients with stable graft function.
	ViKTORIES trial/Lees *et al*. [179]	September 2017–June 2018	90 participants	45 participants	Between-group difference measured by MRI-based ascending aortic distensibility at 12 months	Between-group differences at 12 months of coronary artery calcification score, carotid-femoral pulse wave velocity and augmentation index, MRI measures of cardiac structure and function, office blood pressure, electrocardiogram, calcium metabolism, and bone turnover markers, transplant function, proteinuria, and quality of life.	There was no impact of vitamin K on vascular calcification, vascular stiffness, nor any other outcome measure.

However, due to the lack of absolute evidence on the relationship between vitamin supplementation and cardiovascular health, effective primary CV prevention requires multifactorial control of all major risk factors, as recently demonstrated in the randomized multicenter study NID-2 [180]. The authors suggest that the aforementioned recommendations be put in play, with the due emphasis to be given to the planning and execution of large-scale randomized controlled trials in populations where the actual impact of vitamin supplementation could be monitored thereby helping in establishing the true relationship between vitamins and cardiovascular health.

## Data Availability

Data sharing is not applicable to this article as no datasets were generated or analyzed during the current study.
